# Host Selection, Growth, and Survival of Melonworm (Lepidoptera: Crambidae) on Four Cucurbit Crops Under Laboratory Conditions

**DOI:** 10.1093/ee/nvw067

**Published:** 2016-07-10

**Authors:** B. R. Panthi, D. R. Seal, J. L. Capinera, G. S. Nuessly, C. G. Martin

**Affiliations:** ^1^University of Florida, Tropical Research and Education Center (TREC), 18905 SW 280th St., Homestead, FL 33031 (panthibabu44@gmail.com; dseal3@ufl.edu; cliffgmartin@yahoo.com),; ^3^Department of Entomology and Nematology, University of Florida, Bldg. 970 Natural Area Dr., Gainesville, FL 32611 (capinera@ufl.edu), and; ^4^University of Florida, Everglades Research and Education Center, 3200 E Palm Beach Rd., Belle Glade, FL 33430 (gnuessly@ufl.edu)

**Keywords:** *Diaphania hyalinata*, *Cucurbita*, *Cucumis*, *Citrullus*, host plant

## Abstract

The melonworm, *Diaphania hyalinata* L. (Lepidoptera: Crambidae), is one of the most serious insect problems affecting cucurbit production. We evaluated the relative preference and suitability of yellow squash, zucchini, cucumber, and watermelon to melonworm by measuring its oviposition, larval feeding preference, survivorship, and developmental responses in the laboratory. Whole plants were used for oviposition study, whereas host leaf discs were used for all the other studies. Watermelon feeding resulted in the longest larval development period (14.3 d), greatest prepupal weights and survivals rates (92%; first instar to adult) among the four crops. However, for watermelon, adult oviposition preference (199.5 eggs/♀), egg survival (70%), and larval feeding (4.1% defoliation) were numerically or statistically lowest, and larval head capsule widths and whole-body lengths were smallest. When differences occurred among these variables, yellow squash, zucchini, and cucumber were each typically higher (or quicker to develop) than watermelon. So why do melonworm adults not prefer watermelon, or at least select it as frequently as squash and cucumber when ovipositing? The answer likely is that there might be some variation in the important chemical components among these cucurbits. We suggest that comparison of kairomones and allomones from watermelon and related cucurbits would be very useful for determining the combination resulting in the lowest risk of damage to the more susceptible cucurbits (assuming the levels can be modified without seriously affecting the crops).

Cucurbit crops are widely grown in the United States. In 2014, they collectively yielded about 3.9 million metric tons on 157,370 hectares with a value of US$1.53 billion. In Florida, fresh-market production of cucumber, squash, and watermelon contributed nearly US$247 million to the state economy in 2014 (USDA 2015). The most commonly grown species of cucurbit crops in Florida include *Cucurbita pepo* L. (summer squash and zucchini), *C. moschata* Duchesne ex Lam. (butternut squash and calabaza), *Cucumis sativus* L. (cucumber), *Cucumis melo* L. (cantaloupe or melon), and *Citrullus lanatus* (Thunb.) Matsum and Nakai (watermelon).

Melonworm, *Diaphania hyalinata* L. (Lepidoptera: Crambidae), feeds on plants in the Cucurbitaceae throughout the tropical and subtropical regions of the western hemisphere, but regularly invades all of the southeastern United States (Fulton 1947, Dupree et. al. 1955). It reportedly disperses throughout the southern and Gulf Coast states every summer from southern Florida (Reid et al. 1954, Reid and Cuthbert 1956). Melonworm larvae feed on cucurbit foliage and typically remain on the leaf undersides (abaxial surface). However, when a melonworm population is high, it can devour fruit, leaves, stalks, and vines, leaving only stalk material, veins, and veinlets of leaves (Valles and Capinera 1992). Foliage feeding by melonworm may cause serious damage to host plants (Guillaume and Boissot 2001) and accounts for yield reductions of 9–10% in southern Florida (McSorley and Waddill 1982, Panthi 2015). In southern Florida, melonworms occur throughout the year and cause economic damage to yellow squash, zucchini, cucumber, and watermelon (D.R.S., personal communication, 2014, Panthi 2015).

The ovipositional behavior of adult moths directly affects the development and survival of resulting larvae. Differences in nutritional content and feeding deterrents among host plants may lead to variation in the growth, development, and survival of herbivorous insects like melonworms. These differences can be assessed by measuring larval head capsule widths, pupal dimensions, and other techniques (Dyar 1890, Caltagirone et al. 1983, Godin et al. 2002, Calvo and Molina 2008). Dyar (1890) reported that head capsule widths of Lepidopteran larval instars increase with the number of molts. Other factors influencing larval head capsule size include growing season, generation, parasitism, sex, host-plant cultivar, diet, and temperature (Savopoulou-Soultani and Tzanakakis 1990, Goldson et al. 2001, Frouz et al. 2002). We tested the hypothesis that different cucurbit crops would affect oviposition, larval feeding preference, survivorship, and developmental responses of melonworm. Implications for pest management were also noted.

## Materials and Methods

The study was conducted at the Tropical Research and Education Center (TREC), University of Florida, Homestead, from August to November, 2014.

### Plant Culture

All plants were grown in a greenhouse (11 by 14 m) with the roof blocking 60–70% of the sunlight, hence providing partial shade. Temperatures ranged from 20 to 34°C with an average of 27°C, and relative humidity ranged from 71 to 95% with an average 83%. Four cucurbit crops were used in this experiment: yellow squash, *C. pepo* ‘Enterprise’ (Syngenta Seeds, Pasco, WA); zucchini, *C. pepo* ‘Black Beauty’ (Main Street Seed and Supply, Bay City, MI); watermelon, *C. lanatus* ‘Sugar Baby’ (Main Street Seed and Supply); and cucumber, *C. sativus* ‘Marketmore76’ (Main Street Seed and Supply). All plants were grown by initially planting two seeds 1 cm deep in a 950-ml plastic container (15 cm diameter) filled with a standard artificial soil mixture (Fafard #2, Premier Tech Horticulture, Quakertown, PA). The Fafard #2 mix had ∼70% Canadian peatmoss, 20% perlite, and 10% vermiculite, a pH of 5.5–6.5, and small, unspecified amounts of dolomitic limestone, a wetting agent, and starter nutrients. Newly emerged seedlings were thinned (cut off with scissors) to one plant per container. Plants in each container were irrigated with 50 ml of water twice daily. Granular fertilizer (N-P-K: 8-16-16) at 908 kg/ha was applied to the surface of the artificial medium in each container at planting. Beginning 3 wk after germination, liquid fertilizer (N-P-K: 4-0-8) was also applied weekly through irrigation drips at 236 liters/ha. To prevent infection by fungal pathogens, plants were sprayed and soil was drenched once a week with chlorothalonil (Bravo Weather Stick, Syngenta Crop Protection Co., Greensboro, NC) at 1.75 liters/ha. No insecticides were applied.

### Melonworm Colony Maintenance

The colony was maintained on host plants for two generations before using melonworms in the experiments. The colony was initially started on each host by collecting 100 fourth- or fifth-instar larvae from squash and cucumber plants growing in TREC research plots. Ten to 12 such larvae were placed in a Petri dish (13.5 cm diameter) containing fresh leaves of one or more of the above four cucurbits, which were collected from healthy plants in the greenhouse. Petri dishes containing larvae were then placed in a laboratory room at 28 ± 1.5°C, 77 ± 5% RH, and a photoperiod of 14:10 (L: D) h to allow their development. Petri dishes including leaves were replaced every 24 h with fresh ones to avoid fungal infection. Larvae were checked daily to collect fresh pupae, which were placed in a different Petri dish (8.5 cm diameter) provided with a moist filter paper at the bottom to avoid desiccation. Adults emerging from these pupae reared on a specific host plant cultivar were released into cages with the same host plants to facilitate oviposition, then, newly enclosed larvae were collected from the plants. The same procedures were followed as before until new adults emerged, which were used in conducting different studies on the same host.

### Ovipositional Preference

Ovipositional preference of melonworm was studied in the laboratory room at 28 ± 1.5°C, 77 ± 5% RH, and a photoperiod of 14:10 (L: D) h in a nylon mesh cage (61 × 61 × 183 cm in length, width, and height, respectively). Three cage sides were covered with 200-mesh nylon cloth with a thin plastic sheet covering the remaining side and a zipper on the front providing access to the interior. Four of these cages were used at one time to study ovipositional preference of melonworms and each provided a choice of four cucurbits: yellow squash, zucchini, cucumber, and watermelon. Each cage served as a replication and contained one potted plant (same age) for each of the four host plants resulting in a randomized complete block design. Twenty melonworm adults (28-h-old, unsexed) were collected from the laboratory colony and first immobilized by placing at 7°C for 5–7 min, then added to a Petri dish in the center of each cage equidistant from each plant container. In 4–5 min, the chilled insects became active and started moving to the plants. Within each cage were placed two small glass vials containing 10% sucrose water solution, which served as an adult food and moisture source. Plants were removed 24 h after introducing adults, each plant was carefully inspected, and the number of melonworm eggs recorded. Simultaneously, adult mortality was assessed by gently probing each adult with a fine insect pin: adults not responding by movement were considered dead. Plants were replaced daily with fresh, noninfested same-age plants from the greenhouse. The number of eggs was counted from each plant until all adults in a cage had died.

### Choice and No-choice Tests for Larvae

Two studies were performed under the foregoing laboratory conditions to determine larval melonworm feeding preferences: choice tests and no-choice tests. All larvae (5-d-old second instar) were obtained from the previously described laboratory colony and originated from the same cohort. Leaf discs for choice and no-choice tests were cut from young, fully expanded leaves on greenhouse plants using a 2.5-cm-diameter cork borer with care taken to avoid major leaf veins. For the choice test, twenty 13.5-cm-diameter Petri dishes were used with each dish serving as a replication. Within each dish, a leaf disc from each of the four crop cultivars was placed randomly. One 5-d-old melonworm larva was placed in the center of each Petri dish, which was covered with a lid and left undisturbed. To determine larval preference for each cultivar and the amount of feeding based on leaf area removed, all leaf discs with feeding damage were removed after 17 h, then scanned with a leaf-area meter (LI-COR portable area meter LI 3000, Lambda Instrument Co., Lincoln, NE).

The no-choice test had a 2.5-cm-diameter leaf disc per Petri dish and used four 8.5-cm-diameter Petri dishes, one per cultivar, for each replication. The no-choice study had five replications each time; hence, 20 total Petri dishes were used, which was repeated four times in 10-d study period. Hence, the total replication for each host was 20. Otherwise, experimental procedures were the same as with the choice study.

### Survival and Duration of Eggs

Four of the same nylon mesh cages used to study ovipositional preference were employed in this separate study. Similarly, each cage contained four plants, one for each cultivar, and each plant had its canopy removed except for two leaves, which helped to concentrate oviposition on a minimum number of leaves. Twenty unmated adults (10 males and 10 females), 8–12 h old, were collected from the laboratory colony and placed individually into a plastic vial (6.5 cm high × 2.5 cm diam) with a screen top for ventilation (the preoviposition period is 1–3 d). A wet cotton ball soaked with honey–water (30:70) was placed on the top of the screen cap as a source of nutrition. The plastic vials with melonworm adults were placed in a growth chamber for 72 h at 25 ± 0.5°C, 65–75% RH, and a photoperiod of 14:10 (L:D) h. The adults (80–82 h old) were then transferred to a nylon mesh cage (61 by 61 by 183 cm; length by width by height), and left undisturbed for 4 h (20:00–24:00 EST) to facilitate oviposition on host leaves. Leaves were carefully checked to locate 20 randomly selected eggs (0–4 h old), which were noted by placing red pen marks next to the eggs. Additional eggs on the host leaves were removed with an insect pin. Leaves containing eggs were placed in a Petri dish (15 cm diameter) with a moist filter paper at the bottom to avoid desiccation. Eggs were checked every 4 h to record the embryonic period, which ended with the eclosion of first-instar larvae. Larvae were immediately removed from the Petri dishes to avoid cannibalism, and the number removed per Petri dish per time period was recorded to determine the percentage of larvae eclosing. The study was replicated five times in the laboratory and otherwise used the same procedures as the ovipositional preference study.

### Survival and Duration of Instars

Twenty first-instar larvae (0–2 h old) were collected from the previous test for survival and duration of eggs, then placed individually in a Petri dish (8.5 cm diameter) that contained a 2.5-cm-diameter leaf disc of the same type of crop plant on which the original eggs had been deposited. The larvae were thus from the same cohort. The same procedure was repeated on all four host plants, and the test was replicated five times, which resulted in 20 total Petri dishes. Larvae were examined at 4-h intervals to record molting to the next stage, which was confirmed by the presence of new larval exuviae and by the size and color of head capsules (Smith et al. 1994). Black spots found on the dorsum of each segment identified the larvae as first instars, but for second instars, the spots disappeared. Two white lateral stripes appeared on the dorsum during the third instar, and they became more apparent during the fourth and fifth instars. Once a larva molted, it was taken out of the Petri dish and the time required to molt was recorded. To avoid fungal infection, Petri dishes were replaced with clean ones, and leaves of host plants were replaced daily with fresh leaves. Larvae were transferred from old to new Petri dishes containing fresh leaves by using a clean, sterile paint brush. Once attaining the prepupal stage, larvae lost their two white lateral stripes and stopped feeding. Then, they were placed in clean Petri dishes with moist filter paper on the bottom but without leaves. The filter paper in the Petri dishes functioned as a pupation substrate. Survival and duration of prepupal and pupal stages were also recorded.

### Larval Growth

In a separate study, larval growth was recorded every 2 d to determine the effects of cucurbit cultivar on the development rate of larvae. Twenty first-instar larvae (0–2 h old) originating from the colony for each cucurbit crop were used in the experiment. Each larva was put into a separate Petri dish (8.5 cm diam) that contained a leaf disc (2.5 cm diam) of the same crop cultivar on which the larva originated. Head capsule width and body length of 20 different larvae per host plant in the same age group (cohort) were measured using a microscope with a digital ocular micrometer eyepiece (Leica Application Suite, Leica Microsystems, Wetzlar, Germany). Because larval body length stretches within each stadium and varies with feeding, five measurements were taken for each larva, but only the average of five measurements was used in analyses. In contrast, one measurement was taken for head capsule width of larva. Pupal dimensions were similarly recorded using the same microscope and 20 pupae per treatment were used. The weight of 20 prepupae and pupae per treatment was recorded using an electronic balance (PB3002-S Delta Range, Mettler Toledo, Switzerland).

### Statistical Analysis

All data were square-root transformed for statistical analyses, though only nontransformed data are shown in the tables and figures. Analyses of variance (ANOVAs) for randomized complete block designs were performed for the ovipositional choice test with cages used as blocks (PROC GLM, SAS Institute, 2013). For the duration and survival test of melonworm life stages, cultivar, life stage, and their interaction were first analyzed by factorial ANOVAs (PROC MIXED). One-way ANOVAs (PROC GLM) for completely randomized designs were performed for head capsule width, whole body length, prepupal and pupal measurements, and for choice and no-choice tests. Waller-Duncan K-ratio t-tests were used to compare means for oviposition, survival, and duration of life stages; head capsule widths and whole-body lengths of larvae; prepupal and pupal measurements; and choice and no-choice tests (α < 0.05; Waller and Duncan 1969, SAS Institute 2013).

## Results

### Ovipositional Preference

**Fig. 1.**
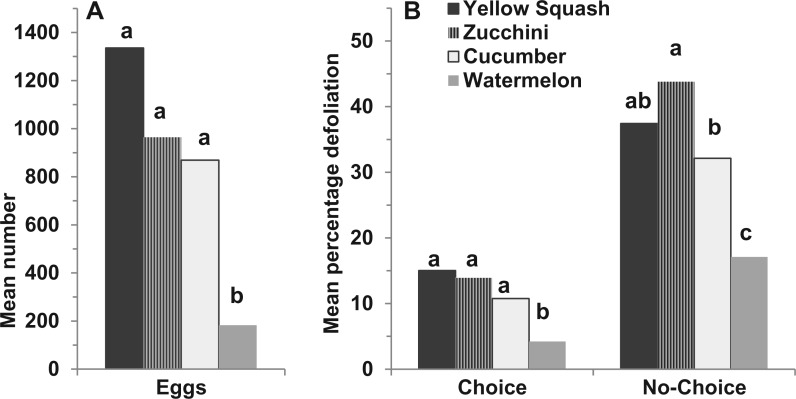
Comparison of *D. hyalinata* preference (or nonrepellence) for different hosts. (**A**) Adult oviposition. (**B**) Larval defoliation (choice and no-choice tests). Means with the same letter did not differ significantly based on a one-way ANOVA followed by a Waller–Duncan K-ratio test (*P = *0.05).

In the laboratory, ovipositional preference of melonworm adults differed significantly among the four cucurbits (*F*_3, 12_ = 11.73, *P* = 0.0007; Fig. 1A). During the adult female life span, significantly fewer eggs were deposited on watermelon than on cucumber, zucchini, or yellow squash. There were no significant differences in oviposition among cucumber, zucchini, and yellow squash.

### Choice and No-choice Tests for Larvae

Larval food-plant cultivars significantly affected the percentage defoliation by melonworm larvae in choice tests (*F*_3, 76_ = 5.64, *P* = 0.0015), with significantly greater levels of defoliation on yellow squash, zucchini, and cucumber than on watermelon (Fig. 1B). The host plant also significantly affected the level of larval defoliation in no-choice tests (*F*_3, 76_ = 12.23, *P* = 0.0002; Fig. 1B). Here, the percentage defoliation was significantly greater on zucchini than on watermelon, with cucumber intermediate between the two, and with yellow squash equal to zucchini and cucumber.

### Survival and Duration

Melonworm survival across all life stages was significantly affected by host plants (*F*_3, 128_ = 8.48, *P* < 0.0001), life stage (*F*_7, 128_ = 9.34, *P* < 0.0001), and the interaction between host plant and life stage (*F*_21, 128_ = 2.33, *P* = 0.002). Egg survival (% eclosion) was significantly greater on yellow squash and cucumber than on zucchini or watermelon (Table 1). In each of the first three instars, the percentage of larvae surviving averaged greater than 81%, and did not differ significantly among host-plant crops. Percentage survival of fourth instars feeding on watermelon was significantly greater than on the other host plants, and larvae feeding on yellow squash had a significantly lower survival rate than those fed on cucumber leaves. Similarly, percentage survival of fifth instars was significantly greater when fed watermelon than yellow squash or cucumber leaves, while those fed cucumber also had significantly lower survival rates than on zucchini leaves. Survival of prepupae and pupae was not significantly affected by which host plant they fed on during their active stages (*P* > 0.05).

**Table 1.** Mean (SE) percentage survival by stage of *D. hyalinata* reared on leaf tissue of yellow squash, zucchini, cucumber, and watermelon in the laboratory

Host	Stage*^a^*							
	Egg	Larva					Prepupa	Pupa
		1st	2nd	3rd	4th	5th		
Yellow squash	89 (4.6)a	93 (2.5)	83 (4.7)	88 (11)	66 (6.4)c	45 (9.5)bc	62 (13)	62 (19)
Zucchini	75 (4.4)b	95 (2.2)	94 (4.0)	97 (2.4)	72 (3.9)bc	65 (7.7)ab	73 (6.0)	50 (7.9)
Cucumber	89 (3.7)a	94 (1)	93 (2.3)	82 (7.0)	80 (2.0)b	32 (8.5)c	45 (16)	37 (15)
Watermelon	70 (5.6)b	91 (1.9)	90 (3.2)	93 (3.1)	96 (2.4)a	91 (3.3)a	93 (4.2)	92 (3.7)
*P**^b^*	**	NS	NS	NS	***	**	NS	NS

*^a^* Means within a column followed by the same letter or no letter did not differ significantly based on analyses of variance followed by Waller–Duncan *K*-ratio *t*-tests (*P* ≥ 0.05).

*^b^* ** *P* < 0.01; *** *P* < 0.001; NS, not significant.

**Fig. 2.**
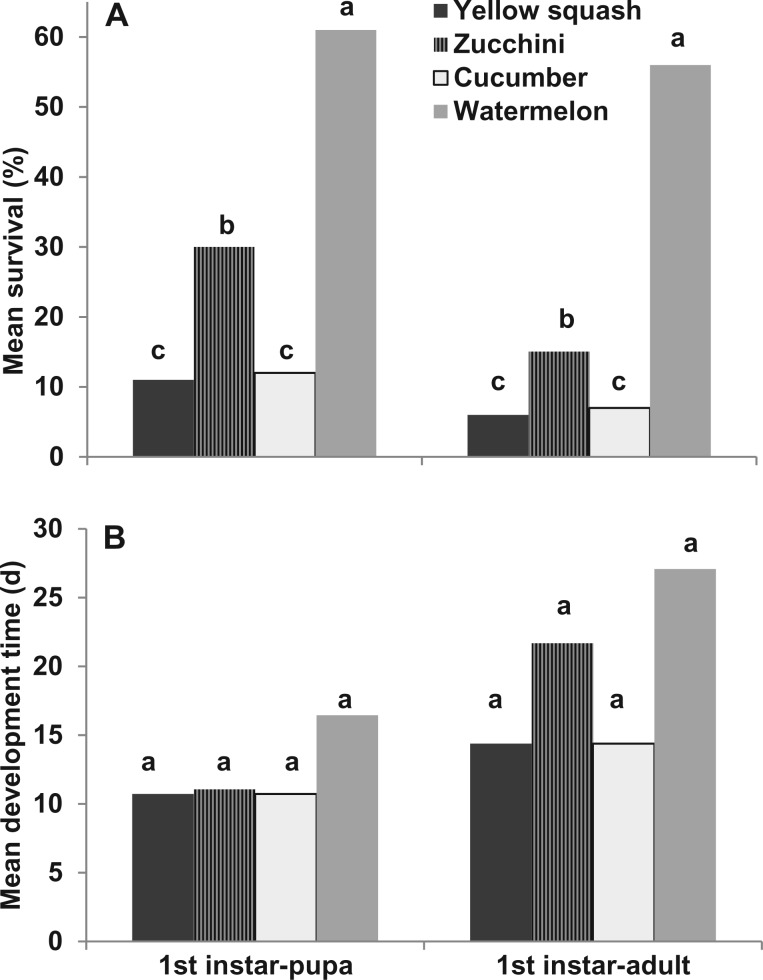
Mean (**A**) percentage survival of larvae and (**B**) developmental time of *D. hyalinata* reared on leaf tissue of four hosts. Means with the same letter did not differ significantly based on a one-way ANOVA followed by a Waller–Duncan K-ratio test (*P* = 0.05).

Host plant significantly affected survival of melonworm across several life stages from first instar to pupa (*F*_3, 16_ = 15.78, *P* < 0.0001) and first instar to adult (*F*_3, 16_ = 20.59, *P* < 0.0001; Fig. 2A). In each case, multistage survival was significantly greater on watermelon than on the other host plants, whereas survival on yellow squash and cucumber was significantly lower than on zucchini or watermelon.

Development times for melonworms across life stages were significantly affected by host plant (*F*_3, 128_ = 3.97, *P* = 0.0096), life stage (*F*_7, 128_ = 36.41, *P* < 0.0001), and the interaction between host plant and life stage (*F*_21, 128_ = 1.85, *P* = 0.0197). However, development times from first instar to pupa and first instar to adult did not vary significantly among host plants (Fig. 2B). Also, there were no significant differences among host plants in egg, first instar, prepupal, or pupal development times (Table 2). However, development times of second, third, and fifth instars were each significantly longer on watermelon than on the other host plants. With fourth instars, development times were significantly longer on watermelon than on zucchini or cucumber. Development times for third and fourth instars were significantly shorter on zucchini than on the other host plants.

**Table 2.** Mean (SE) development time (d) by stage of *D. hyalinata* reared on leaf tissues of yellow squash, zucchini, cucumber, and watermelon in the laboratory

Host	Stage*^a^*							
	Egg	Larva					Prepupa	Pupa
		1st	2nd	3rd	4th	5th		
Yel. squash	4.3 (0.1)	3.1 (0.1)	2.1 (0.1)b	1.9 (0.1)b	1.7 (0.1)ab	2.0 (0.2)b	2.1 (0.1)	8.4 (2.1)
Zucchini	4.7 (0.2)	3.0 (0.1)	2.2 (0.1)b	1.4 (0.0)c	1.3 (0.1)c	1.2 (0.1)b	1.9 (0.2)	11 (0.2)
Cucumber	5.1 (0.4)	3.1 (0.0)	2.1 (0.0)b	2.0 (0.0)b	1.6 (0.1)b	1.8 (0.5)b	1.7 (0.5)	6.2 (2.5)
Watermelon	4.2 (0.1)	3.3 (0.1)	2.9 (0.1)a	2.5 (0.2)a	1.9 (0.0)a	3.7 (0.2)a	2.4 (0.1)	11 (0.2)
*P**^b^*	NS	NS	***	***	***	**	NS	NS

*^a^* Means within a column followed by the same letter or no letter did not differ significantly based on analyses of variance followed by Waller–Duncan *K*-ratio *t-*tests (*P* ≥ 0.05).

*^b^* ** *P* < 0.01; *** *P* < 0.001; NS, not significant.

### Growth

Head capsule widths of melonworm larvae were significantly affected by host plant (*F*_3, 128_ = 20.83, *P* < 0.0001), larval age (*F*_7, 128_ = 806.19, *P* < 0.0001), and the interaction of host plant with larval age (*F*_21, 128_ = 3.69, *P* < 0.0001). Larval head capsules were significantly wider on yellow squash than on watermelon at 5, 7, 9, 11, and 13 d after emergence (Table 3). Head capsules of 5-d-old larvae reared on cucumber and 7-d-old larvae from zucchini were both significantly narrower than those reared on yellow squash, but significantly wider than on watermelon. Larvae reared on zucchini had significantly greater head capsule widths than larvae on watermelon 5, 7, 9, and 13 d after emergence. There were no significant differences between larvae reared on zucchini and watermelon 11 d after eclosion; between yellow squash and zucchini 5, 9, or 13 d after eclosion; or between yellow squash and cucumber 7, 9, 11, or 13 d after eclosion. Also, head capsule widths did not differ significantly among the host plants for 1-, 3-, and 15-d-old larvae.

**Table 3.** Mean (SE) head capsule width (mm) of *D. hyalinata* larvae reared on leaf tissue of yellow squash, zucchini, cucumber, and watermelon in the laboratory

Host*^a^*	Days after eclosion*^b^*							
	1	3	5	7	9	11	13	15
Ys	0.3 (0.0)	0.3 (0.0)	0.46 (0.03)a	0.72 (0.03)a	1.2 (0.1)a	2.1 (0.1)a	2.0 (0.0)a	2.1 (0.0)
Zu	0.3 (0.0)	0.3 (0.0)	0.48 (0.01)a	0.65 (0.02)b	1.3 (0.1)a	1.5 (0.1)b	2.1 (0.0)a	2.0 (0.0)
Cu	0.3 (0.0)	0.3 (0.0)	0.39 (0.02)b	0.72 (0.03)ab	1.2 (0.1)ab	1.8 (0.2)ab	2.1 (0.0)a	2.1 (0.1)
Wa	0.3 (0.0)	0.3 (0.0)	0.31 (0.01)c	0.46 (0.02)c	0.9 (0.0)b	1.4 (0.1)b	1.7 (0.2)b	2.1 (0.0)
*P**^c^*	NS	NS	***	***	*	**	*	NS

*^a^* Ys (Yellow squash), Zu (Zucchini), Cu (Cucumber), Wa (Watermelon).

*^b^* Means within a column followed by the same letter or no letter did not differ significantly based on analyses of variance followed by Waller–Duncan *K*-ratio *t-*tests (*P* ≥ 0.05).

*^c^* * *P* < 0.05; ** *P* < 0.01; *** *P* < 0.001; NS, not significant.

Body lengths of melonworm larvae were significantly affected by host plant (*F*_3, 128_ = 18.94, *P* < 0.0001), larval age (*F*_7, 128_ = 769.94, *P* < 0.0001), and the interaction of host plant and larval age (*F*_21, 128_ = 3.12, *P* < 0.0001). Host plant significantly affected body length 1, 5, 7, and 9 d after eclosion (Table 4). Body lengths of 1-d-old larvae reared on zucchini were significantly longer than those of yellow squash or watermelon. Body lengths for 5-, 7-, and 9-d-old larvae reared on zucchini, yellow squash, and cucumber were significantly longer than on watermelon. There were no significant differences among host plants for body lengths in 3-, 11-, 13-, or 15-d-old larvae. There also were no significant differences in the length, width, or weight of pupae among melonworms reared on yellow squash, zucchini, cucumber, or watermelon (Table 5). However, prepupal weights were significantly greater on watermelon and cucumber than on yellow squash.

**Table 4.** Mean (SE) body length (mm) of *D. hyalinata* larvae reared on leaf tissue of yellow squash, zucchini, cucumber, and watermelon in the laboratory

Host*^a^*	Days after eclosion*^b^*							
	1	3	5	7	9	11	13	15
Ys	1.5 (0.1)b	2.7 (0.2)	4.7 (0.6)a	8.5 (1.1)a	14 (1.2)a	18.1 (0.3)	26.2 (1.3)	26.7 (1.2)
Zu	2.0 (0.2)a	3.0 (0.1)	4.9 (0.3)a	8.5 (0.4)a	15.4 (1.4)a	18.1 (0.9)	27.6 (0.6)	24.3 (1.6)
Cu	1.7 (0.1)ab	2.8 (0.2)	3.9 (0.5)a	7.4 (0.2)a	15.0 (0.8)a	18.0 (0.7)	28.9 (0.8)	26.7 (0.7)
Wa	1.4 (0.2)b	3.1 (0.3)	2.6 (0.1)b	4.5 (0.5)b	9.1 (0.3)b	16.3 (0.9)	23.3 (0.7)	26.6 (0.8)
*P**^c^*	*	NS	**	***	***	NS	NS	NS

*^a^* Ys (Yellow squash), Zu (Zucchini), Cu (Cucumber), Wa (Watermelon).

*^b^* Means within a column followed by the same letter or no letter did not differ significantly based on analyses of variance followed by Waller–Duncan *K*-ratio *t-*tests (*P* ≥ 0.05).

*^c^* * *P* < 0.05; ** *P* < 0.01; *** *P* < 0.001; NS, not significant.

**Table 5.** Mean (SE) length (mm), width (mm), and weight (mg) for prepupal and pupal *D. hyalinata* reared on leaf tissue of yellow squash, zucchini, cucumber, and watermelon in the laboratory

Host	Weight (g)*^a^*		Pupal dimensions (mm)*^a^*	
	Prepupa	Pupa	Length	Width
Yellow squash	0.07 (0.01)b	0.06 (0.01)	16.6 (0.56)	3.83 (0.24)
Zucchini	0.08 (0.01)ab	0.07 (0.00)	17.24 (0.47)	3.77 (0.09)
Cucumber	0.09 (0.01)a	0.06 (0.00)	17.34 (0.28)	3.71 (0.08)
Watermelon	0.10 (0.01)a	0.07 (0.01)	18.06 (0.33)	3.81 (0.07)
*P**^b^*	**	NS	NS	NS

*^a^* Means within a column followed by the same letter or no letter did not differ significantly based on analyses of variance followed by Waller–Duncan *K*-ratio *t-*tests (*P* ≥ 0.05).

*^b^* ** *P* < 0.01; NS, not significant.

## Discussion

Oviposition preference by melonworm adults was examined in a choice test comparing numbers of eggs deposited per host plant. In these studies, melonworm deposited more eggs on yellow squash, zucchini, and cucumber than on watermelon. Hence, adults appeared to prefer watermelon the least among the four host plants. Preferences were also determined for larvae in choice and no-choice tests from percentages of defoliation per host plant, and similarly showed that yellow squash, zucchini, and cucumber were preferred over watermelon. But because the survival of melonworm larvae was highest when fed watermelon foliage, it is not a poor host for melonworm. Consumption of watermelon foliage was less than in the other hosts, perhaps indicating a high level of nutrition in watermelon. Hence, this may suggest why greater defoliation was observed on other cucurbits than on watermelon. Melonworm females reportedly deposit more eggs on leaves of yellow squash because of the presence of nonvolatile, highly polar, amphoteric compounds with relatively low molecular weights (Peterson et al. 1994, Peterson and Elsey 1995). Allelochemicals are biologically produced, nonnutritional chemicals that have interspecific effects; specifically, kairomones benefit the receiver and allomones benefit the producer (Whittaker and Feeny 1971). In Cucurbitaceae, cucurbitacins are widely distributed chemicals which may act as allomones, because they are deterrents to many foliage-feeding insects. However, for *Diabrotica* spp. (Coleoptera: Chrysomelidae), cucurbitacins are phagostimulants and serve as kairomones (Whittaker and Feeny 1971, Bernays and Chapman 1994). The presence of a greater number of eggs on leaves of yellow squash than on watermelon may also have resulted from ovipositional repellents (allomones) on watermelon leaves, though these are not known to occur. Physical differences (e.g., leaf color, texture, trichome density) may also explain the lower level of oviposition on watermelon, but likewise has not been studied.

Although the most preferred host plant for oviposition was yellow squash, it yielded lower survival rates for fourth-instar larvae than cucumber or watermelon and for fifth instars than watermelon. Similar differences in survival were also found in tests of first instar to pupa survival, and first instar to adult survival: survival rates on watermelon were greater than on zucchini, which yielded greater rates than yellow squash or cucumber. The greater prepupal weights found on watermelon are consistent with the survival rate assessment, suggesting that watermelon was the superior host plant.

Apparently contrasting with the foregoing survival results were the differences found in development times per life stage, hence allowing different implications on fitness. Development times per life stage were usually longer for larvae on watermelon than on the other host plants. Similarly, when differences were present between host plants, the head capsule widths and body lengths were usually smaller for larvae on watermelon than on the other host plants. Herbivorous insects increase in abundance, growth, and reproduction when fed a nutrient-rich diet (Auclair et al. 1957, Dixon 1970, Weibull 1987, Sandstrom and Pettersson 1994). According to Dyar (1890), food nutrients can affect the growth and development of insect larvae, which can be measured by head capsule widths. In addition to the slowest development of larvae occurring on watermelon leaves, head capsule widths from watermelon may have been reduced by low nutrient content (Savopoulou-Soultani and Tzanakakis 1990). When measuring head capsule widths, the larvae originated from eggs, collected on same day (and thus were of the same cohort), to minimize effects of varying number of days between measurements per instar. Therefore, our differences in head capsule widths, body lengths, and dimensions and weights of pupae seemed to reflect differences in treatments (host plants). The greater survival rates and prepupal weights of melonworms raised on watermelon than on the other host plants seemed to indicate greater fitness on watermelon. However, some of the observed variables supported reduced fitness on watermelon compared with one or more of the other cultivars. Also, differences between yellow squash, zucchini, and cucumber tended to be less frequent and/or less conclusive than between these plants and watermelon.

Of the four cucurbits tested, yellow squash, zucchini, and cucumber are the most common and suitable hosts for melonworm, whereas watermelon is a minor host (Capinera 2008). Presumably, melonworm females choose the host plant and locate their eggs according to the food suitability for resulting larvae, and an environment that permits maximum fitness. However, watermelon is as suitable as squash and cucumber based on larval and pupal weights, and is superior to squash and cucumber based on survival. So why do melonworm adults not prefer watermelon, or at least select it as frequently as squash and cucumber when ovipositing? The answer likely is that insect host preference usually is based on coevolution with wild plants, not human-bred cultivars. Melonworm behavior is founded on chemical stimuli that have allowed it to best survive with wild plants, and watermelon is not a natural host; thus, it is an anomaly. We hypothesize that there are three possible explanations for this situation: 1) the chemical stimuli (kairomones) used by melonworm to identify suitable host plants (cucurbits) were naturally lower or absent in the primitive plants that eventually were selected to produce watermelon; 2) the chemical stimuli attractive to ovipositing moths have been inadvertently diminished by plant breeders as they selected for horticulturally improved watermelon plants; and 3) less likely, but also possible, is inadvertent incorporation of oviposition deterrents into watermelon breeding lines. Presently, we do not know with certainty the basis for reduced oviposition on watermelon. However, comparison of the kairomones and allomones originating from watermelon and related cucurbits may result in valuable information for reducing the risk of damage to the more susceptible cucurbits (assuming the levels can be modified without seriously affecting the crops). Future studies should focus on testing a larger number of cucurbit taxa (both wild and cultivated) for melonworm ovipositional preference and development. In addition, assessment of the 15 chemical constituents of the cucurbits would help to better understand the mechanisms for host suitability.
